# A dual-channel visible light optical coherence tomography system enables wide-field, full-range, and shot-noise limited human retinal imaging

**DOI:** 10.1038/s44172-024-00167-7

**Published:** 2024-01-31

**Authors:** Jingyu Wang, Stephanie Nolen, Weiye Song, Wenjun Shao, Wei Yi, Amir Kashani, Ji Yi

**Affiliations:** 1 Department of Ophthalmology, Johns Hopkins University, Baltimore, MD 21231 USA; 2 Department of Biomedical Engineering, Johns Hopkins University, Baltimore, MD 21231 USA; 3 Department of Medicine, Boston University School of Medicine, Boston Medical Center, Boston, MA 02118 USA

**Keywords:** Applied optics, Optical imaging

## Abstract

Visible light optical coherence tomography (VIS-OCT) is an emerging ophthalmic imaging method featuring ultrahigh depth resolution, retinal microvascular oximetry, and distinct scattering contrast in the visible spectral range. The clinical utility of VIS-OCT is hampered by the fundamental trade-off between the imaging depth range and axial resolution, which are determined by the spectral resolution and bandwidth, respectively. To address this trade-off, here we developed a dual-channel VIS-OCT system with three major advancements including the first linear-in-K VIS-OCT spectrometer to decrease the roll-off, reference pathlength modulation to expand the imaging depth range, and per-A-line noise cancellation to remove excess noise, Due to these unique designs, this system achieves 7.2 dB roll-off over the full 1.74 mm depth range (water) with shot-noise limited performance. The system uniquely enables >60° wide-field imaging which would allow simultaneous imaging of the peripheral retina and optic nerve head, as well as ultrahigh 1.3 µm depth resolution (water). Benefiting from the additional near-infrared (NIR) channel of the dual-channel design, this system is compatible with Doppler OCT and OCT angiography (OCTA). The comprehensive structure-function measurement enabled by this dual-channel VIS-OCT system is an advance towards adoption of VIS-OCT in clinical applications.

## Abstract (web summary)

Wang, Nolen and colleagues present an iteration of the existing visible light optical coherence tomography system, which improves both imaging depth, resolution and suppresses noise. The system was demonstrated in human retinal imaging.

## Introduction

Since its conception in the 1990s, optical coherence tomography (OCT) has been one of the most influential imaging methods in biophotonics and has revolutionized clinical practice in ophthalmology^1^. Visible light optical coherence tomography (VIS-OCT) is a burgeoning modification of OCT that uses visible instead of near-infrared (NIR) illumination and has been demonstrated for preclinical and clinical ophthalmic imaging^2–4^. One advantage of VIS-OCT is that visible wavelengths demonstrate higher levels of backscattering from biological tissues than longer near-infrared wavelengths^5^ and reveal distinct spectral contrast in imaging pigmentation (e.g. rhodopsin in photoreceptors, melanin^6,7^, hemoglobin^8–10^). More importantly, the shorter central wavelengths of visible light spectra result in improved transverse resolution and, when bandwidth is maintained, improved axial resolution^11^. This enhanced resolution allows detailed imaging of structures such as Bruch’s membrane^12,13^, sub-bands in the photoreceptor outer segments^14^, sub-layers of the inner plexiform layer^15^, and the texture of nerve fibers^13^. Another key application of VIS-OCT is microvascular oximetry in the retina, which leverages the strong oxygen-dependent absorption spectrum of hemoglobin in the visible range^16,17^. By performing 3D segmentation and spatio-spectral analysis, VIS-OCT specifically extracts signals within blood vessels to calculate the blood oxygen saturation^8,18,19^ and, furthermore, the oxygen metabolic rate when incorporating Doppler blood flow measurements^19^.

There are still challenges in VIS-OCT that must be addressed for broader clinical utility. The most fundamental challenge arises from the trade-off between imaging depth range and axial resolution. Current VIS-OCT systems use spectral domain (SDOCT) or Fourier domain (FDOCT) configurations with a linear line scan camera to record the interferogram. While the full potential of VIS-OCT requires a broad bandwidth to achieve wide spectral contrast and better axial resolution, this proportionally worsens the spectral resolution (unit: nm/pixel) thus reducing the total imaging depth range. The limited depth range is further exacerbated by the sensitivity roll-off over depth^20^, a common characteristic in FDOCT systems. The roll-off is particularly problematic in broadband FDOCT because of the non-linear distribution of wavenumbers over the pixels^21^, resulting in spectrally dependent roll-off that compromises the total performance^20,22,23^. To improve spectral resolution and imaging depth, line cameras with high pixel numbers can be used^24^, with the caveat of slower imaging speed and challenging optical design to account for aberration over a long linear pixel array. The limited depth range is particularly cumbersome in the presence of involuntary eye movements that occur during imaging of awake subjects, as well as in wide-field imaging outside the macula where large retinal curvatures are present^25^.

To overcome this fundamental trade-off in VIS-OCT, we have developed this device that incorporates several designs. First, to overcome the spectrally dependent roll-off, we developed the first linear-in-K VIS-OCT spectrometer covering a 140 nm bandwidth (500–640 nm). Second, we implemented reference pathlength modulation to achieve full-range OCT. This doubles the total imaging depth range and corrects retinal curvature for wide-field imaging. Third, to eliminate the excess noise at the zero-delay line due to the supercontinuum generation, we implemented per-A-line noise cancellation in the visible channel to achieve shot-noise limited imaging.

By the above designs, we have achieved in total 7.2 dB roll-off (~50% improvement over the state-of-the-art VIS-OCT spectrometer) over a full range of 1.74 mm imaging depth (water), with a depth resolution down to 1.3 μm (water). We demonstrate the following: a wide-field capable VIS-OCT (retinal imaging >60˚ viewing angle in the eye, which is the largest so far); full-range circular scanning at the optic nerve head (ONH); OCT angiography (OCTA) by the NIR-OCT channel; and, finally, high-definition, ultrahigh resolution retinal imaging. Human imaging interaction with the device is improved by minimizing visible light exposure through a rapid line rate of 120 kHz. The system design is a critical step forward to improve clinical utility, and the imaging capability could potentially generate impactful knowledge by revealing structural and functional changes in retinal and/or neurodegenerative pathologies.

## Results

### Overview of system design

**Fig. 1:**
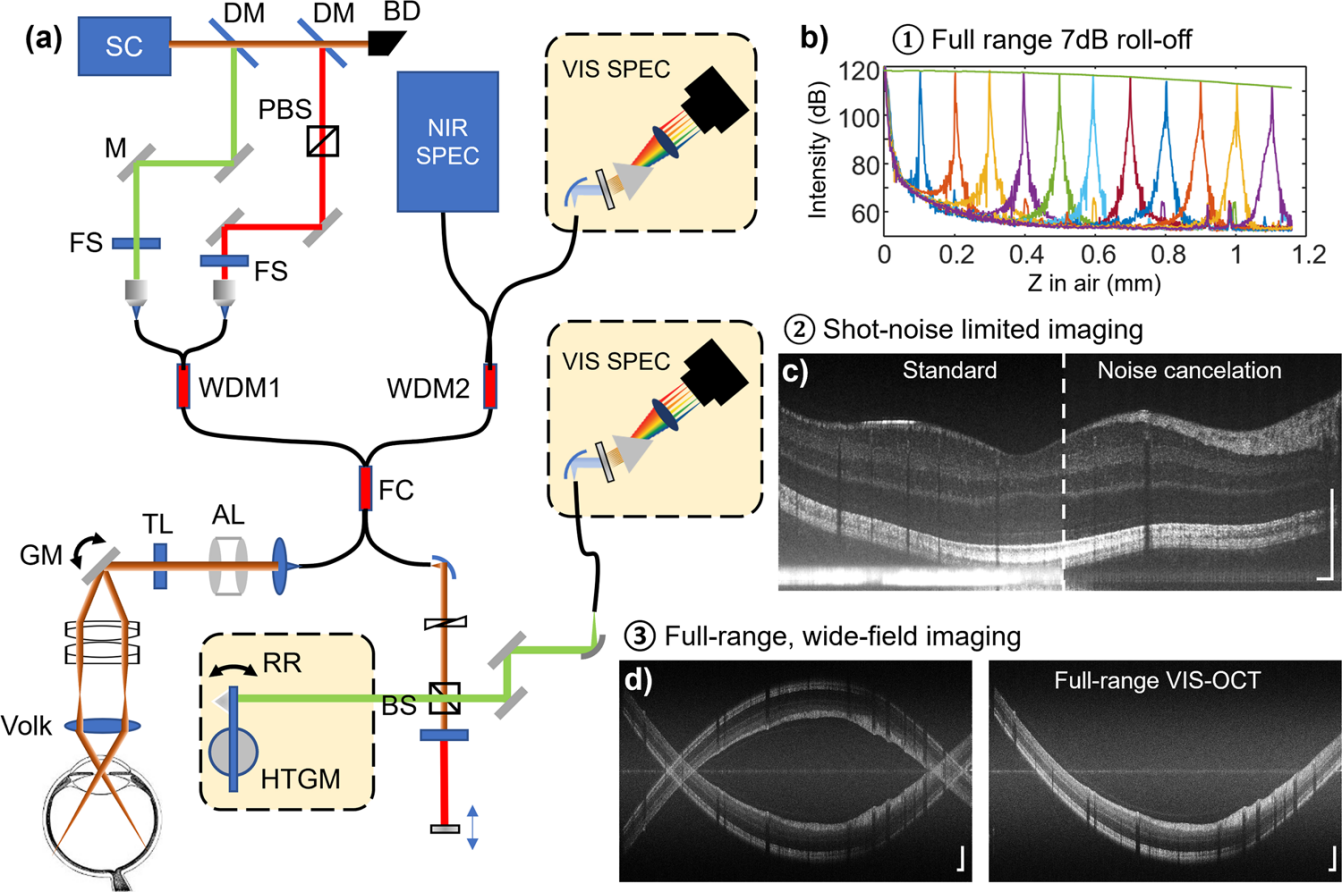
Overview of the dual-channel VIS-OCT. Left: (**a**) schematic of the optomechanical configuration. Three key enabling designs for full-range, wide-field imaging are the (1) linear-in-K VIS-OCT spectrometer; (2) per-A-line noise cancellation by a second spectrometer; and (3) reference pathlength modulation. Right: summary of the imaging performance by three optical designs, achieving (**b**) 7.2 dB roll-off over the entire imaging range; (**c**) shot-noise limited performance; and (**d**) doubling of the imaging depth range. DM dichroic mirror, SC supercontinuum source, BD beam dump, PBS polarization beam splitter, FS filter set, WDM wavelength division multiplexer, FC fiber coupler, AL achromatizing lens, TL tunable lens, RR retroreflector, HTGM high torque galvanometer, BS beam splitter. Scale bars: 0.2 mm.

The configuration of the dual-channel VIS-OCT is shown in Fig. 1a. Dual-channel imaging was implemented by two wavelength division multiplexers (WDMs) to combine and split the visible and NIR bands, and an ultra-broadband fiber coupler forming a Michelson interferometer. The two channels can operate alone or simultaneously and complement each other. VIS-OCT can provide ultrahigh resolution imaging and retinal oximetry, while NIR-OCT is used for initial alignment, OCT angiography (OCTA), and Doppler OCT. The dual-channel broadband light is provided by a supercontinuum source with two filter sets to output a visible band (500–650 nm) and an NIR band (750–900 nm). To account for the chromatic shift over the large bandwidth, a custom-designed achromatizing lens (AL)^26^ was installed in the sample arm. In addition, a tunable lens was used for focal adjustment. The detailed components and optics are provided in the method section. The depth resolution is 1.3 and 3.7 microns in water for VIS-OCT and NIR-OCT, respectively. The powers at the cornea are <0.22 and <0.9 mW in VIS-OCT and NIR-OCT, respectively. All data shown here were acquired at a 120 kHz A-line rate.

**Fig. 2:**
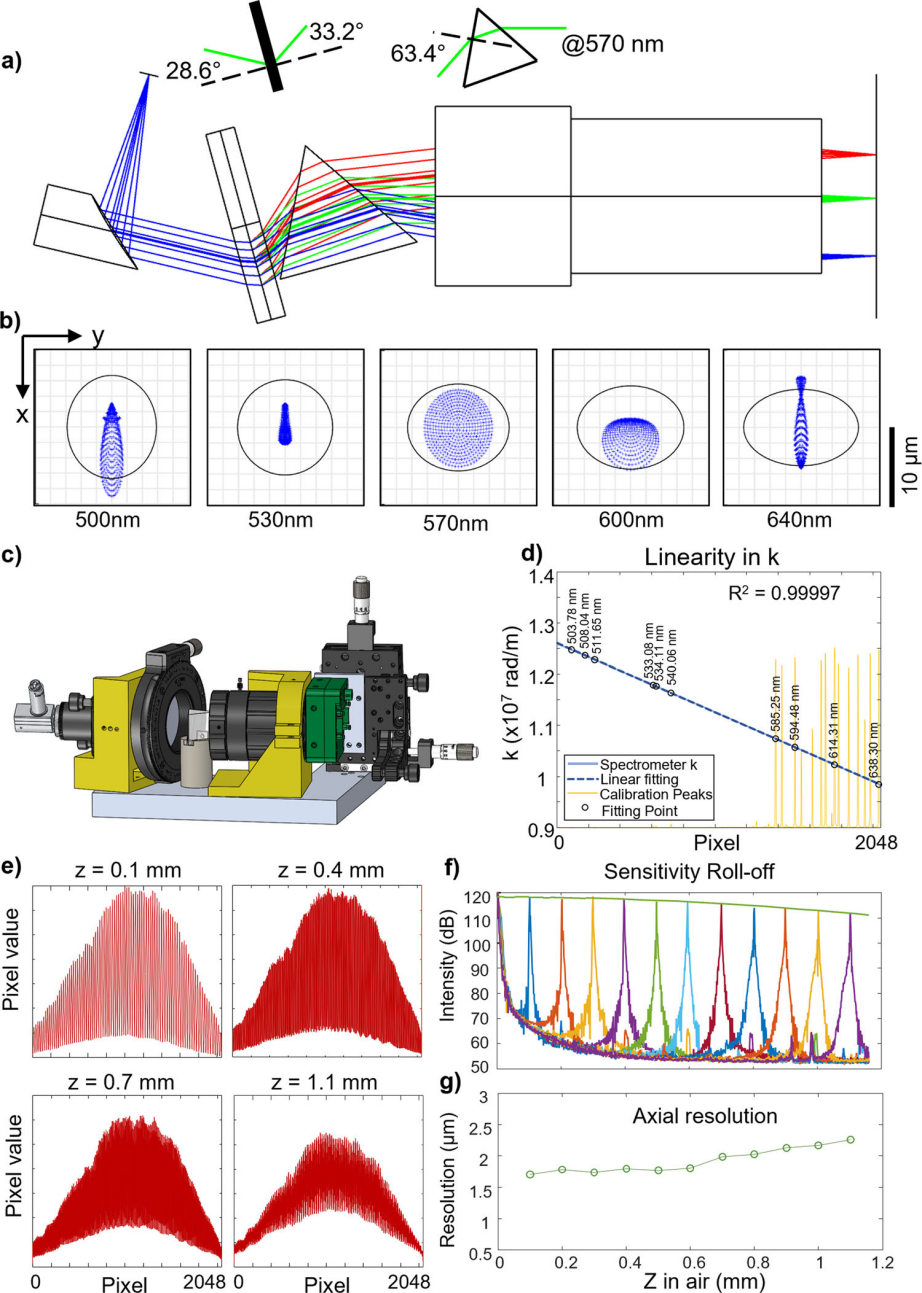
Design and characterization of linear-in-K VIS-OCT spectrometer. **a**–**c** The optical layout, the spot diagrams in Zemax at different wavelength over the spectral range, and the 3D model of the spectrometer. Three colors in (**a**) traced 500, 570, and 640 nm respectively. **d** Spectrometer calibration using a Neon lamp, and the corresponding characteristic peaks. Pixel index is highly linear to the wavenumber, *k*, with R^2^ = 0.99997. **e** The raw interferogram spectra by two mirror reflections with varying optical delay. **f**–**g** Sensitivity roll-off and axial resolution characterization over the entire imaging depth range.

The major effort of this paper is to overcome the trade-off between the spectral bandwidth, axial resolution, and imaging depth in VIS-OCT. To this end, three key design components are highlighted in Fig. 1: (1) Linear-in-K spectrometers that eliminate the spectrally dependent roll-off (Fig. 1b); (2) Noise cancellation to suppress the excess noise to enable high-contrast and shot-noise limited imaging (Fig. 1c); and (3) Reference pathlength modulation that achieves full-range imaging, doubling the imaging depth while maintaining the same resolution (Fig. 1d). The corresponding components are highlighted in Fig. 1a and explained in detail in the following sections. Scanning protocols for all figures are summarized in Table 1 in “Methods” section.

### Linear-in-K VIS-OCT spectrometer

The design principle of a linear-in-K spectrometer has been described in the literature^22^, and is first achieved here for VIS-OCT. Figure 2a shows the Zemax model, consisting of a collimator (*f* = 50 mm), a transmission grating (Wasatch Photonics, 1800 lines/mm), an equilateral dispersive prism (Thorlabs, F2), a focusing lens (Edmund Optics, CA series, *f* = 75 mm), and a line scan camera (e2V, Octoplus 2048, 250 kHz). The prism introduces a nonlinear dispersion that results in a linear-in-K relationship when the light is focused on the camera pixels. We performed a Zemax simulation with spot diagrams at 5 wavelengths, showing that the diffraction-limited resolutions were achieved (Fig. 2b). The 3D rendering model including all the mechanical components is shown in Fig. 2c. The mountings in yellow were 3D-printed, while the line scan camera was mounted on a 5-axis stage with x-y-z translation and yaw-pitch angle adjustment for precise alignment of the camera sensor. We calibrated the spectrometer using a Neon lamp and confirmed excellent linearity (R^2^ > 0.999) in wavenumber k = 2π/λ, where λ is the wavelength (Fig. 2d). The exact wavelength coverage is from 498.9 to 639.3 nm. We next evaluated the roll-off performance of the linear-in-K spectrometer using a reflective mirror to provide the sample signal. The interferogram fringe amplitudes at varying depths are shown in Fig. 2e. The contrast of the fringe decreased with the increasing depth, yet the change is rather consistent over the entire bandwidth (*i.e*. independent of wavelength), a distinct benefit of the linear-in-K spectrometer. The fringe contrast is slightly tapered off at the edge of the bandwidth at the deeper range, presumably due to the field curvature of the focusing lens (Supplemental Fig. 5). We characterized the overall roll-off performance in Fig. 2f and measured 7.2 dB decay by polynomial fitting over the entire depth range of 1.16 mm in air and 0.87 mm in water. After implementing full-range imaging, the imaging depth can be doubled to 2.32 mm (air) and 1.74 mm (water) with the same roll-off decay. This total roll-off over the entire imaging depth is ~50% that of the state-of-the-art conventional VIS-OCT spectrometer at ~14-15 dB (Supplemental Table 1). Full-width-half-maximum (FWHM) width from the mirror surface was used to characterize the axial resolution in Fig. 2g, down to ~1.7 μm (air) and 1.3 μm (water) close to the DC delay line. The resolution is maintained within 2.3 μm (air) and 1.7 μm (water) throughout the entire depth range.

### Per-A-line noise cancellation by simultaneous reference signal collection

All current VIS-OCT systems use supercontinuum (SC) light sources. However, the nonlinear phenomena lead to power fluctuations in the spectra^27^ known as excess noise^28^, which are added on top of the shot (Poisson) noise caused by the quantum nature of photons^29^. Noise can be reduced by increasing the spectrometer exposure time, essentially averaging out the noise fluctuations^29^, while also increasing the imaging time. When imaging at higher speeds, a better noise minimizing technique is the balanced detection method^28,30–32^. By leveraging the temporal signature of excess noise and calibrating two spectrometers with sub-pixel precision^33^, recent work showed that the excess noise background can be effectively removed in spectral domain OCT^32^.

**Fig. 3:**
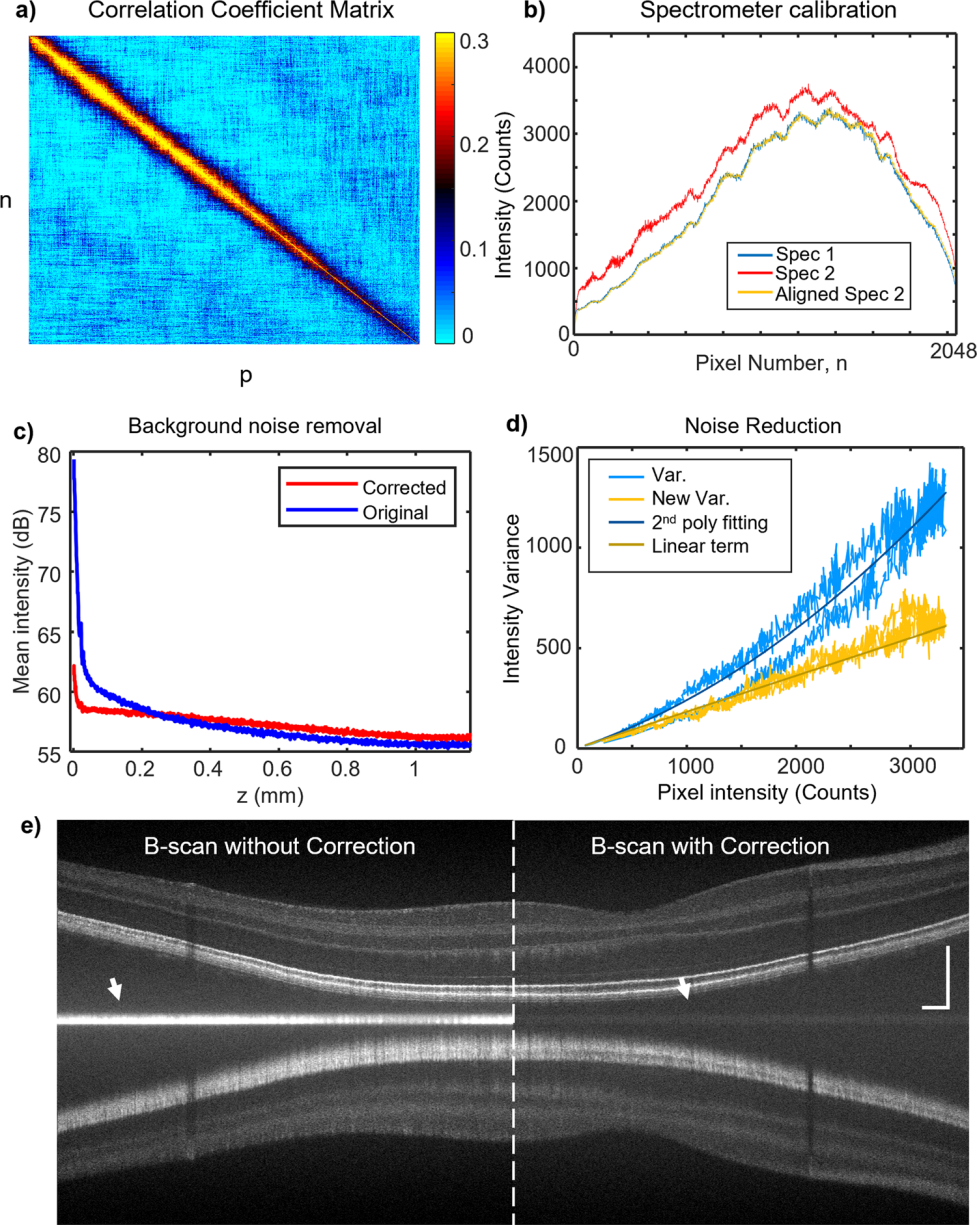
Per-A-line noise cancellation. **a** Pixel-pixel cross-correlation matrix over continuous acquisition between two VIS-OCT spectrometers. n, first spectrometer; p, second spectrometer. **b** Spectrum match before and after spectrometer alignment. **c** Background noise in spatial domain before and after noise cancellation. **d** Intensity variance per intensity value over all pixels before and after noise cancellation. **e** Example of B-scans with and w/o noise cancellation. Scale bar: 0.2 mm.

We implemented noise cancellation by simply splitting the reference signal to a second identical VIS-OCT spectrometer (Fig. 1a) using the excess noise correlation method proposed by Kho et al^33^. Two spectrometers were triggered simultaneously, acquiring temporal spectral signals, ${x}_{1}\left(n,T\right)$, ${x}_{2}\left(p,T\right)$, where *n*, *p* = 1–2048 denote camera pixel index, and *T* denotes discrete time stamps. Continuous acquisition allows calculation of pixel-wise temporal correlation between two spectrometers, shown in Fig. 3a. We further calculated the correlation coefficient matrix after noise cancellation, which appeared as an almost diagonal matrix, indicating independence among pixels, a characteristic of shot noise and dark noise (Supplemental Fig. 7). The maximum values of correlation coefficients were used for pixel calibrations, resulting in a sub-pixel alignment precision (Refer to corresponding Methods section for detailed processes) and a close spectral matching after calibration/scaling (Fig. 3b).

We took 512 acquisitions of ${x}_{1}\left(n,T=1-512\right)$ and generated a conventional background B-scan image. The DC components were removed by subtracting the mean spectrum, ${x}_{1}\left(n,T\right)-\bar{{x}_{1}\left(n\right)}$. The averaged A-line signal is then shown in Fig. 3c, where RIN produced a large noise background at zero-delay depth. In comparison, by per-A-line noise cancellation, we removed the DC components by ${x}_{1}\left(n,T\right)-{x}_{2}^{{\prime} }\left(n,T\right)$, where ${x}_{2}^{{\prime} }\left(n,T\right)$ is the calibrated/scaled spectra from the second spectrometer. The averaged A-line over the same 512 acquisitions reduces the background at the zero-delay depth by ~4-fold (Fig. 3c). Toward deeper imaging depth, the noise floor with cancellation is higher than without cancellation, due to the additional shot noise by the second spectrometer^32^. We further characterized the noise statistics by plotting the temporal variance σ^2^ for all 2048 pixels versus their mean pixel value (blue curve in Fig. 3d). For the original ${x}_{1}\left(n,T\right)$, a quadratic term is present when fitting the data with a second order polynomial, representing the excess noise^34^. We then plotted the variance for ${x}_{1}\left(n,T\right)-{x}_{2}^{{\prime} }\left(n,T\right)$, versus the same mean pixel values (yellow curve in Fig. 3d). It matches well with only the linear term from the same polynomial fitting, representing a Poisson distribution and a shot-noise limited performance. The benefit of noise cancellation is clearly shown in an example VIS-OCT image in Fig. 3e, where the bright background in the zero-delay depth is suppressed to a faint gray shade as indicated by the white arrows. With noise cancellation introduced, the roll-off is further improved by ~1 dB (Supplemental Fig. 6). We noticed some residual noise floor at the DC line after the noise cancellation that is not perfectly removed. The noise cancellation is a critical step in achieving the following full-range VIS-OCT, since the zero-delay line lies in the middle of the image.

### Wide-field and full-range VIS-OCT imaging

**Fig. 4:**
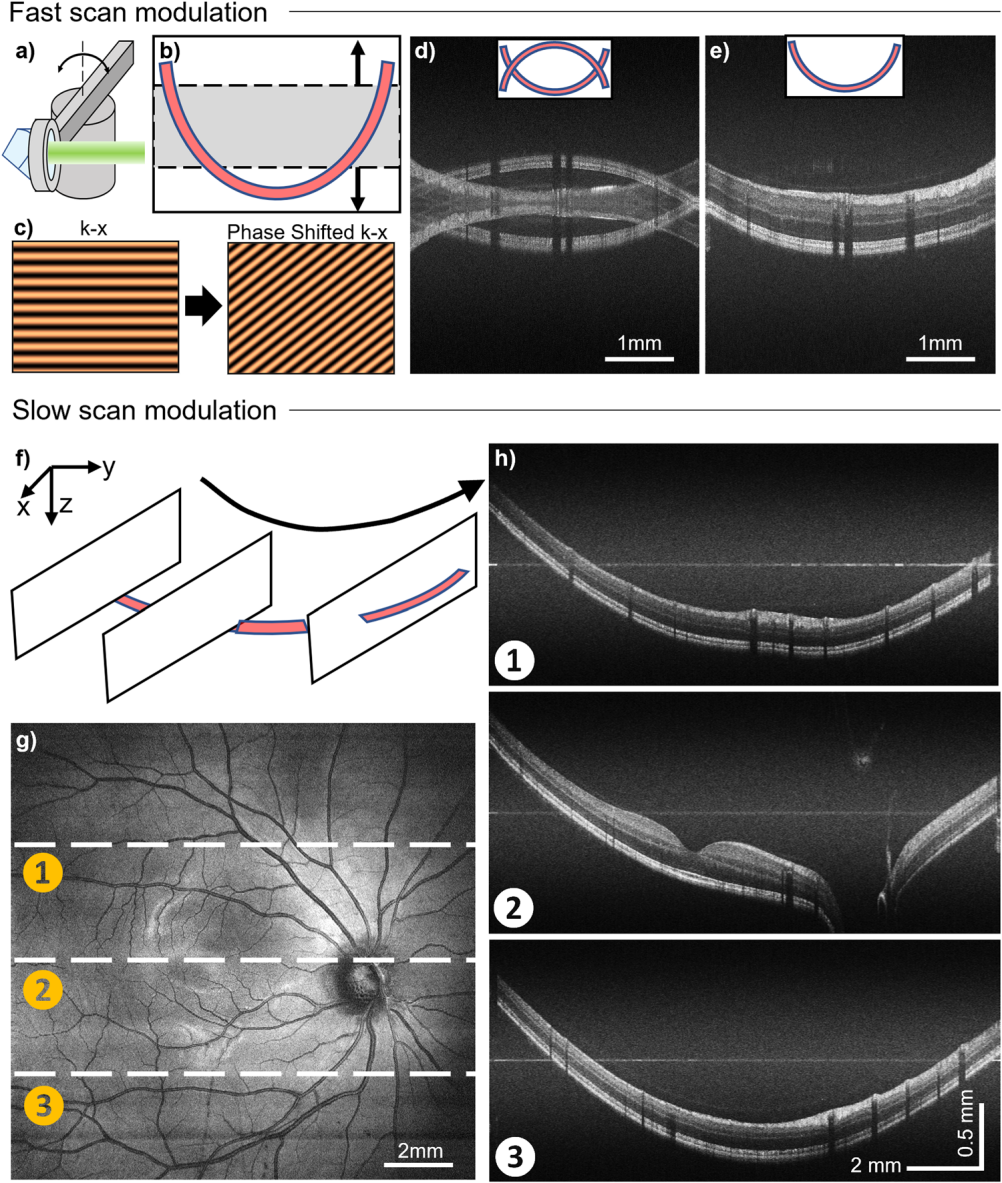
Full-range and wide-field VIS-OCT enabled by the reference pathlength modulation. **a**, **b** Schematic model of the retroreflector (RR) and high-torque galvanometer (HTGM) to modulate the reference pathlength. **c** The phase shift introduced by pathlength modulation to extend the imaging depth by fast scan modulation. **d**, **e** The comparison between conventional VIS-OCT and full-range VIS-OCT to remove the Fourier transform ambiguity. **f** Schematic for slow scan modulation to compensate for the retinal curvature by adjusting the reference path per B-scan. **g**, **h** Wide-field VIS-OCT en face projection and its corresponding full-range VIS-OCT B-scans from locations in (**g**).

When studying regions outside of the macula, it is necessary to have sufficient imaging depth to account for eye curvature. To address the trade-off between imaging resolution and depth, and to enable wide-field VIS-OCT imaging, we implemented reference pathlength modulations. The hardware setup was accomplished by mounting a retroreflector on a high torque galvanometer (HTGM)^25^ as depicted in Fig. 4a. The rotation of the shaft modulates the reference pathlength. For the fast scan modulation (Fig. 4b), the retroreflector moves slowly at a constant speed, introducing a phase modulation into the *k*-x domain within the B-scan (Fig. 4c), where *x* denotes the fast axis. A Hilbert transform can be performed along *x* to obtain complex interferogram values^35^. This phase modulation removes the phase ambiguity when performing a Fourier transform, so that the OCT image symmetric to the zero-delay line can be eliminated and the imaging range is doubled. Successful removal of the mirror image is evident when comparing a typical VIS-OCT image (Fig. 4d) with full-range imaging (Fig. 4e). The full-range imaging protocol requires oversampling for phase modulation. We used 16x A-line density compared to the raster scan, and decreased B-scans proportionally to maintain the imaging time (2.6 s) for human subjects.

Within the same raster scanning, the slow scan modulation also permitted wide-field retinal imaging by moving the zero delay of the image in an arc corresponding to the eye curvature (Fig. 4f). The range of slow scan modulation only depends on the travel range of HTGM in the order of several millimeters. The en face image in Fig. 4g has corresponding cross sections in Fig. 4h from three B-scan locations. The position of the retina is maintained in each full-range B-scan image, mitigating the typical displacement of the retina caused by the eye curvature. Meanwhile, the curvatures within each B-scan frame are entirely covered due to the extended imaging depth range (~1.7 mm in retina) (Fig. 4h). The optics allow a > 60˚ viewing angle to image the previously unattainable peripheral retina (Supplemental Fig. 1).

**Fig. 5:**
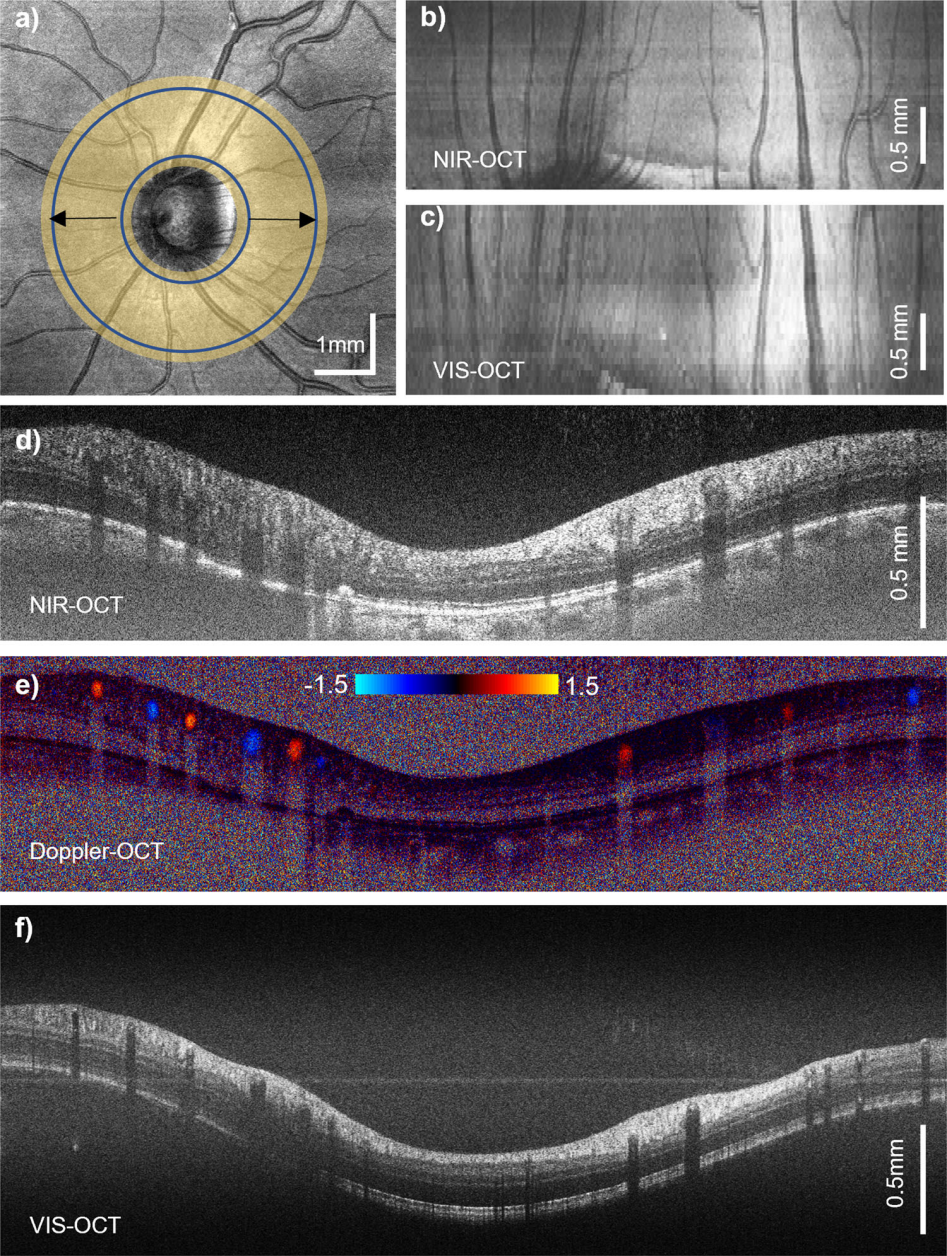
Full-range VIS-OCT imaging at the optic nerve head (ONH). **a** En face projection of a VIS-OCT scan centered at ONH. Shaded area shows the scan area by circular scanning with increasing radius. **b**, **c** Expanded scan area samples all major vessels in peripapillary region. **d**–**f** Simultaneous acquisition of NIR-OCT, Doppler OCT and VIS-OCT by circular B-scan pattern shown in previous panels.

The extended depth range is particularly advantageous in imaging the optic nerve head (ONH), the peripheral retina, or a staphyloma, where large curvatures are present. We designed a scanning pattern where a series of dense circular B-scans (4096 Aline per B scan) were repeated with an increasing radius around the ONH, covering a 0.8–2.5 mm radius around the ONH (Fig. 5a). The expanded *en face* projection from VIS-OCT and NIR-OCT scans are shown in Figs. 5b and 5c, intersecting all major vessels in the retinal circulation exiting and entering the ONH. The dense circular scan can serve two purposes at the same time: (1) to enable Doppler OCT, by calculating the phase delay between two adjacent A-lines in the NIR OCT channel^36^; and (2) to allow the fast scan modulation in the VIS-OCT channel for full-range imaging. Figure 5d, e exemplify circular scans in NIR-OCT and the corresponding phase contrast for Doppler OCT. We note that the NIR channel is preferable to VIS-OCT for Doppler OCT applications due to the better penetration through large vessel lumen. Meanwhile, Fig. 5f demonstrates that circular full-range VIS-OCT easily covers the curvature of the parapapillary retina within the frame, which would otherwise be clipped.

### High-definition dual-channel structural imaging

**Fig. 6:**
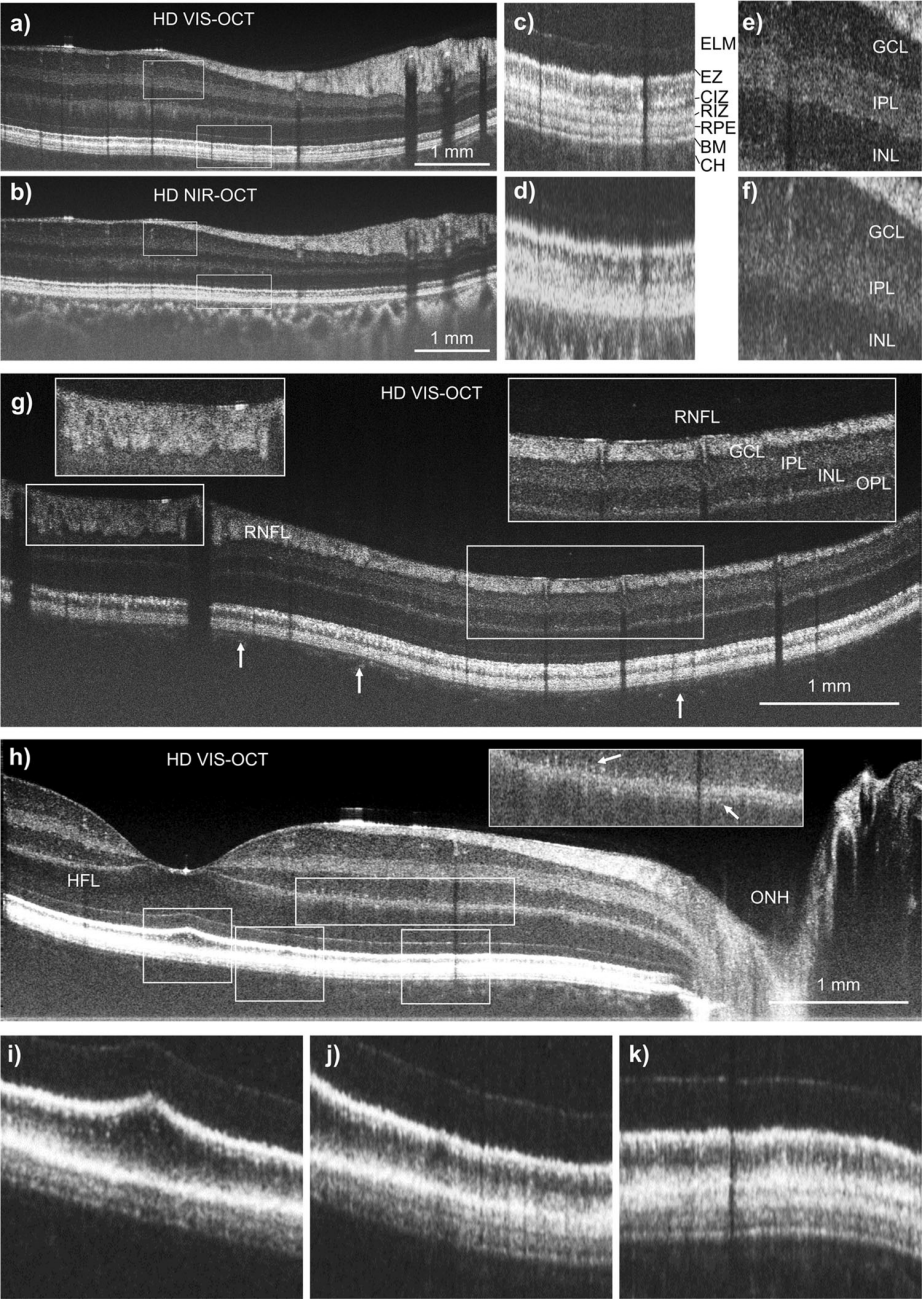
High-definition (HD) VIS-OCT reveals features of retinal sub-layer structure. Comparison of HD VIS-OCT (**a**, **c**, **e**) and HD NIR-OCT (**b**, **d**, **f**) B-scan images, with high-magnification images showing retinal sub-layers resolvable by HD VIS-OCT. ELM external limiting membrane, EZ ellipsoid zone, CIZ cone interdigitation zone, RIZ rod interdigitation zone, RPE retinal pigment epithelium, BM Bruch’s membrane, CH choroid, GCL ganglion cell layer, IPL inner plexiform layer, INL inner nuclear layer. **g**, **h** Additional retinal sub-layer features using HD VIS-OCT including RNFL texture, OPL capillaries, and OPL sub-layers where putative synaptic structures between photoreceptors and bipolar cells would occur. The contrast was adjusted in (**h**) to better define Henle’s layer in the outer retina. **i**–**k** Zoomed in images on the outer segment of photoreceptors in (**h**).

By addressing the imaging range in VIS-OCT, we can fully leverage the high axial resolution enabled by the broadband linear-in-K VIS-OCT spectrometer. We adopted a speckle reduction protocol^37^ and averaged 16 A-lines at each pixel to generate high-definition (HD) imaging. Figure 6a, b displays the simultaneously acquired HD VIS-OCT and HD NIR-OCT images. The depth resolution in the NIR channel is estimated to be ~3.7 microns in water based on the reference spectrum. The penetration into the choroid in VIS-OCT is weaker than in NIR-OCT due to stronger absorption by the retinal pigment epithelium (RPE) and choroidal pigment. The improvement of the resolution in HD VIS-OCT is visually apparent with finer anatomical stratification than HD NIR-OCT. High magnification images of two areas in the outer segment of the photoreceptors and the inner plexiform layer (IPL) demonstrate resolution of previously undescribed retinal features (Fig. 6c–f). HD VIS-OCT clearly resolved at least six bands from the external limiting membrane (ELM) to Bruch’s Membrane (BM), including the putative IS/OS junction, putative cone outer segment tip (COST), putative rod outer segment tip (ROST), RPE, and BM^14^ (Fig. 6c). There are also numerous sub-bands resolvable in VIS-OCT in the IPL, potentially corresponding to different synaptic connections between bipolar cells and retinal ganglion cell dendrites^3,15^ (Fig. 6e). High magnification images of the retinal nerve fiber layer (RNFL) and ganglion cell layers (GCL) from the HD VIS-OCT illustrate previously unresolvable features within the fiber bundle, as well as a clear, thin GCL (Fig. 6g). Although the penetration into the choroid is limited in VIS-OCT, we are still able to image the signal beyond the choriocapillaris in the perifoveal and parafoveal areas (arrows in Fig. 6g). Figure 6h shows interesting structural details in the outer plexiform layer (OPL) where the photoreceptors presumably synapse with bipolar cells. The deep capillary plexus in the retinal circulation is visible, noted by the hyperreflective signals above the OPL. We intentionally adjusted the contrast in Fig. 6h to show the Henle’s fiber layer (HFL) as a lighter band above the slightly more hyperreflective outer nuclear layer (ONL). Beyond the better anatomical imaging enabled by the VIS-OCT, the signal contrast may also be distinct between VIS-OCT and NIR-OCT due to the wavelength differences, suggesting potential for scattering spectroscopic analysis^24,26,38^. Figure 6i-k further shows the different bands in the outer segment of the photoreceptors. At the fovea pit, rods are most abundant and the ROST is the dominant band above the RPE (Fig. 6i). The COST starts to appear at the perifoveal region below the ROST (Fig. 6j) and becomes a well-separated band further towards the ONH (Fig. 6k).

### OCTA imaging

**Fig. 7:**
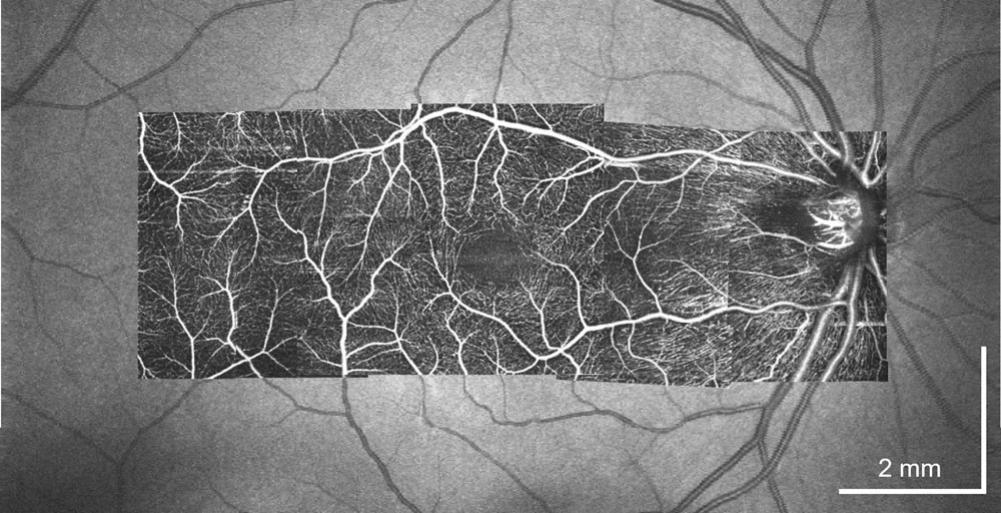
OCT angiography (OCTA) by the NIR channel showing capillary level microvasculature. The montage was generated from 5 separate acquisitions. Each OCTA image covers a 3.3 × 3.3 mm FOV.

The dual-channel configuration of this device is compatible with clinical NIR-OCT and OCT angiography (OCTA). Figure 7 demonstrates an en face montage OCTA covering a range of 13.3 × 7.4 mm overlaid on a wide-field NIR-OCT scan. The OCTA is composed of five separate acquisitions, each with ~50% overlapping area with the adjacent acquisition, showing the major vessels as well as the single-capillary level network from internal limiting membrane (ILM) to OPL.

## Discussion

In this paper, we presented the dual-channel VIS-OCT device that offers three features including (1) unparalleled roll-off performance with a linear-in-K spectrometer, (2) extended imaging range up to 1.74 mm in tissue at 1.3–1.7 microns depth resolution, and (3) a wide-field imaging capability from the reference pathlength modulation. Implementing noise cancellation achieves shot-noise limited performance, a key step in removing the excess noise near the zero-delay line for full-range VIS-OCT imaging. We show that the dual-channel system not only offers micron-level ultrahigh resolution VIS-OCT images but is also compatible with Doppler OCT and OCTA using the NIR channel. The practical advantage of the NIR channel is rapid image alignment, a major challenge in implementing VIS-OCT in clinics.

### Further technical refinements

The spectrometer is the key component in VIS-OCT, given the stringent power safety limit and relatively smaller imaging depth range compared to those of NIR-OCT. The linear-in-K design eliminates the spectrally dependent roll-off and notably improves the overall imaging performance compared to the conventional designs. The optical design enables diffraction-limited resolution smaller than the 10 μm pixel size in the camera, and thus the roll-off is determined solely by the pixel averaging. The additional refractive prism may introduce a slight light loss at the two prism-air surfaces; however, the Fresnel refraction is polarization-dependent. The light loss can be eliminated by adding a polarization controller before spectrometer. We also should note that the axial resolution in VIS-OCT can be further improved by tuning the spectral shape to be a top-hat instead of a semi-Gaussian, such that the spectrometer bandwidth can be fully utilized.

The noise cancellation in this presented form is a straightforward add-on to the existing Michelson interferometry, simply splitting and recording a fraction of the reference light. The previous methods used balanced detection, typically implemented in swept-source OCT (SSOCT) systems, with a Mach–Zehnder interferometer by subtracting two simultaneous interferograms with a phase shift of π to cancel out excess noise and improve the signal to noise ratio^28,32^. While the balanced detection scheme still uses a line-scan camera without gains as in SSOCT, the doubled dynamic range offered by the second camera does provide a signal boost. To this end, the balanced detection approach for per-A-line noise cancellation is considered a better configuration in principle. The full benefit of balanced detection in VIS-OCT would still need to wait for a swept-source operating in visible light range. Balanced detection in SSOCT allows an increase in the dynamic range of the interferogram signal due to analog subtraction of the DC signal, and subsequent analog gain in the balanced photodetector circuit. This would allow further sensitivity improvement than the digital balanced detection in SDOCT.

### Potential clinical applications

This dual-channel VIS-OCT enables unparalleled capability to study structure/function relations in the retina for a broad range of potential clinical applications. The ultrahigh resolution reveals structural details that are unattainable with conventional OCT imaging. For example, improved characterization of RNFL, GCL, and IPL sub-layers have direct clinical implications in quantifying damage in glaucoma and Alzheimer’s disease, as well as other optic neuropathies. In the outer retina, VIS-OCT images provide a tool to understand the origins of hyperreflective bands in the outer segment of photoreceptors^39–42^ and will provide unparalleled resolution of pathological changes in diseases such as age-related macular degeneration, Plaquenil toxicity, and central serous retinopathy.

Another attractive potential of the system is the extensive analysis of retinal perfusion including measurement of blood oxygen saturation (sO_2_)^8^, Doppler blood flow in major arterioles and venules^19^, and OCTA at the capillary level^2,9^. Visualization of retinal sub-layers and capillaries will provide avenues of research to understand the interaction of ischemia and neurodegeneration in diabetes as well as other ischemic retinopathies such as central retinal vein occlusion, choroid neovascularization, etc.

Simultaneous acquisition of VIS-OCT and NIR-OCT images can provide spectroscopic information about retinal features that will be associated with different pathologies. For example, a “true color” 3D reconstruction of a mouse retina using RGB reconstruction in VIS-OCT was reported to examine the spectral contrast in photoreceptors, RPE, and retinal lesions^38,43,44^, where photo-sensing pigments and melanin play important roles maintaining physiological conditions. Similar analysis by VIS-OCT has been performed in human retinae by contrasting the VIS-OCT and NIR-OCT images^4,24,26^. The spectroscopic analysis may reveal subtle structural contrasts determined by local refractive index fluctuations, serving as sensitive markers for nanoscale changes beyond the resolution limit. For example, reflectance spectral analysis has suggested cytoskeleton disruption in RGC axons and has shown promising clinical applications in early glaucoma detection^4^.

In conclusion, we have developed a dual-channel VIS-OCT system to address the fundamental trade-off between image depth range and resolution. We achieve unparalleled roll-off performance so far for VIS-OCT (7.2 dB over 1.74 mm in retina), a wide field-of-view (>60°) and shot-noise limited imaging. The comprehensive imaging capabilities enabled by dual-channel design can be an important tool in studying structure/function relations in retinal pathologies.

## Methods

### System setup and control

The VIS (500 to 650 nm) and NIR (750–900 nm) illumination were initially selected from a supercontinuum light source (Superk EXTREME, EXU-OCT-6, NKT Photonics) by two dichroic mirrors (DMLP650R and DMLP900R, Thorlabs). Two bandpass filter sets in VIS and NIR channels further filtered the light (FEL0500 + FES0650 in VIS channel, and FEL0750 + FES0900 in NIR channel, Thorlabs). An electrical shutter (#87-208, Edmund Optics) was put in the VIS channel for alignment purposes. The filtered light of the dual-channel was coupled into a wavelength division multiplexer (WDM 560/830, Customized, Thorlabs) to become the input of a 90/10 fiber coupler (FC, TW670R2A2, Thorlabs). The output end (10%) of the FC was connected to the sample arm and the other end (90%) was directed to the reference arm. In the sample arm, the light beam passed through a collimator (*f* = 6 mm, #63-714, Edmund Optics), customized achromatizing lens^26^, tunable lens (TL, EL-10-30-TC-VIS-12D, Optotune), two-axis galvanometer scanner (GVSM002, Thorlabs), and 3:1 telescope (*f* = 75 mm, and *f* = 25 mm, Volk 40D). In the reference arm, before the light enters a beam splitter (BS), it traverses a collimator (*f* = 15 mm, RC04APC-P01, Thorlabs) and a series of in the optical path. The 50:50 beam splitter (BS016, Thorlabs) worked with two edge filters (FES0700 and FEL0750, Thorlabs) to separate the VIS and NIR beam to a retroreflector (Edmund Optics, #48-605) and a reference mirror, respectively. The retroreflector and the reference mirror were mounted on a HTGM (Nutfield, QS20) and a translational stage, respectively. The light reflected from the reference mirror returned through the optical path and was separated by a second WDM, then was recorded by a commercial spectrometer (Cobra-S 800, CS800-850/140, Wasatch Photonics) for NIR and a customized linear-in-K spectrometer for VIS-OCT. For noise cancellation, a fraction of the light reflected from the retroreflector and transmitted through the beam splitter was coupled into a separate single mode fiber and collected by a second VIS-OCT linear-in-K spectrometer. The raw spectra in both channels are shown in supplemental Fig. 2. The maximum light power on the pupil was controlled to be <0.22 mW (VIS) and <0.9 mW (NIR) which is safe under the ANSI standard of ophthalmic instrument. We characterized the sensitivity in our system which was 90.5 dB using a specular surface^45^. A silver mirror was put in the sample arm to generate the interferogram. We put an ND filter (OD = 2) in front of the mirror to introduce the attenuation. The power before and after the ND filter was 238 µW and 2.9 µW. The exposure time for sensitivity characterization was 9.1 µs. The optical path difference between the sample and reference arms was around 100 µm. After the accurate dispersion compensation, the sensitivity was calculated.

We set up two LED displays of 24x8 MAX7219 dot matrix modules on each side of the imaging path in front of a subject’s eyes as external fixation targets. The displays were controlled by an Arduino Uno R3 microcontroller with a joystick. The control program was developed in the Arduino IDE (v1.8.19). Only one dot in each display was illuminated and controlled by the moving command of the joystick. The photograph of the packaged device is shown in supplemental Fig. 3. We used three frame grabbers (two PCIe-1437 and one PCIe-1433, National Instruments) to receive the data from three line scan cameras (OctoPlus, Teledyne e2v), which were triggered by the I/O device (USB-6363, National Instrument). The I/O device also controlled the electric shutter in the VIS-OCT channel, 2D galvanometers, and HTGM. We developed a customized software in Microsoft Visual Studio 2019 combining Qt (v5.15.2), QCustomPlot (v2.1.0)^46^, FFTW (v3.3.5)^47^, NI-DAQmx (v21.0.0), and NI-IMAQdx (v21.0.0) using C/C + + for the preview and acquisition. Multithreaded programming was used to acquire the data in preview and acquisition modes for the three cameras and in parallel real-time image display in the preview. We achieved a frame rate of 20 fps (VIS) and 15 fps (NIR) with a simultaneous preview of 4 frames to show the B-scan of the retina for alignment. Several imaging protocols used in this study were pre-defined and selected for acquisition.

### Linear-in-K spectrometer design and configuration

Two custom built linear-in-K spectrometers were used for detection in the visible light channel. The spectrometer setup, similar to various near-infrared designs^22,48–50^, used a diffraction grating and a prism to respectively diffract and refract the incoming light into linear-in-K spacing. After light collimation, the beam must pass through a diffraction grating whose incident angle (θ_i_) for the first order is calculated as:

1 $${\theta }_{i}=\arcsin \left(\frac{{\lambda }_{0}N}{2}\right)$$

Where λ_0_ is the central wavelength and N is the number of lines per meter (1800 line/mm). The diffracted angle (θ_d_) for a given wavelength λ_m_ about the central wavelength is given by:

2 $${\theta }_{d}=\arcsin \left({\lambda }_{m}N-\sin \left({\theta }_{i}\right)\right)$$

Letting θ_i_ = θ_d_, we resulted in θ_i_ = θ_d_ = 28.6 degrees at 532 nm per the grating manufacturer’s specifications. Since the diffracted wavelengths about the center wavelength are approximately linear by wavelength, an equilateral dispersive prism is added to change the distribution. For the angle of minimum deviation, the prism incidence and deviation angles are equal. Using Snell’s Law and prism geometry, and assuming the refractive index of air is unity, the prism incident angle for the minimum deviation path (θ_pi_) at a chosen wavelength (λ_1_) is given by:

3 $${\theta }_{{pi}}=\arcsin \left({n}_{1}\sin \left(\frac{{60}^{\circ }}{2}\right)\right)$$

where n_1_ is the index of refraction of the prism at for λ_1_. For the center wavelength of 570 nm, the value for θ_pi_ = 63.4°. Using the geometry of the prism, and combining the prism dispersion with the diffraction grating, the output angle from the spectrometer can be found from the previous equations as:

4 $${\theta }_{{out}}=\arcsin \left({n}_{m}\sin \left({60}^{\circ }-\arcsin \left(\frac{\sin \left(\left({\theta }_{d}-{\theta }_{i}\right)+{\theta }_{{pi}}\right)}{{n}_{m}}\right)\right)\right)$$

Where n_m_ is the refractive index of the prism glass at a given wavelength λ_m_. Substituting *k*_m_ = 2π/λ_m_ yields a nearly linear relationship between wavenumber and distance on the spectrometer’s camera after passing through a fixed focal length lens.

The theoretical design was first simulated in OpticStudio (Zemax) to compare the spot size of several wavelengths to the pixel size of the line camera. The spectrometer was then constructed using an 1800 line per mm volume phase holographic grating (WP-1800/532-50.8, Wasatch Photonics) and an F2 glass dispersive prism (PS852, Thorlabs). A 75 mm CA series fixed focal length lens objective (11-321, Edmund Optics) focused the dispersed light onto a 2048-pixel line camera (OctoPlus EV71Y01CCL2210-883, Teledyne e2v) with a linear-in-K distribution.

### Per-A-line noise cancellation VIS-OCT imaging

Two spectrometers were used to provide per-A-line noise cancellation. Using a beam splitter cube, part of the visible reference signal was directly transmitted to the second visible light spectrometer. After simultaneous acquisition from two spectrometers ${x}_{1}\left(n,T\right)$ and ${x}_{2}\left(p,T\right)$, pixel calibration was performed using the spectral encoding property of the excess noise of the supercontinuum^33^. The specific temporal noise patterns were used to calibrate the two spectrometers through a correlation matrix defined by the correlation of the spectrometer pixels during a shared collection period. For each pixel in *x*_*1*,_ the most correlated pixel in *x*_*2*_ was found by locating the maximum correlation. The pixel correspondence of n and p was fitted by a second-order polynomial, which was then used to interpolate the second spectrometer data ${x}_{2}\left(p,T\right)$ to ${x}_{2}^{{\prime} }\left(n,T\right)$ to align the two devices. After alignment, pixel-wise scaling factors δ(n) were calculated by δ(n) = ${x}_{1}\left(n,T\right)$ / ${x}_{2}^{{\prime} }\left(n,T\right)$, to match the two spectra. The variables of the second-order polynomial and scaling factors were stored.

For noise cancellation VIS-OCT imaging, the DC components were removed by subtracting ${x}_{2}^{{\prime} }\left(n,T\right)$ after the interpolation and scaling, followed by the regular OCT image processing methods, including digital dispersion compensation and Fourier transform. Since the spectrometer is already linear-in-K, spectral interpolation for linear k-spacing is not required.

### Full-range wide-field OCT with reference modulation

A high torque galvanometer scanner (Nutfield, QS20) with an attached retroreflector was used to provide full-range wide-field images. This setup was used to replace a stationary reference arm mirror and provide modulation of the reference pathlength. Two different modulation strategies were implemented in fast and slow scanning directions.

The fast scanning modulation achieves full-range imaging. Since the intensity signal of VIS-OCT is real, the Fourier transform of the signal is Hermitian, and the image has mirror terms centered on the zero-delay line. The wavenumber interferogram signals from each A-line can be represented by the signal *I(k, x)*, where *k* is the wavenumber and *x* is the lateral A-line locations within the B-scan.

5 $$I\left(k,x\right)={I}_{s}+{I}_{r}+2\sqrt{{I}_{s}\left(k,x\right){I}_{r}\left(k\right)}\cos \left[k\Delta L\left(x\right)\right]$$

*I*_*s*_ and *I*_*r*_ are the intensities from the sample and reference arm respectively, and ΔL is the pathlength difference. For the sake of simplicity, we neglect *I*_*s*_ and *I*_*r*_ and only keep the third interferogram term in the following equations. Due to the nature of the retroreflector, the direction of the incoming beam is reversed without a change in angle, directly changing the path length difference. Previous full-range modulation methods typically rely on a piezo translation stage^35,51^ or introducing a beam offset in the scanning mirror^52–54^. We applied the same linear ramping voltage pattern to the HTGM that introduced an approximately linearly changing reference path length,

6 $$\Delta L\left(x\right)=L+x\,\delta L$$

where $\delta L$ is the pathlength increment (unitless) introduced by the HTGM. Then the modulated interferogram can be rewritten as

7 $${I}^{{\prime} }\left(k,x\right)=2\sqrt{{I}_{s}\left(k,x\right){I}_{r}\left(k\right)}\cos \left[{kL}+k\delta L\bullet x\right]$$

A Hilbert transform to the x dimension was used to extract the complex version of the *k-x* interferogram as,

8 $$\widetilde{H}\left(k,x\right)=2\sqrt{{I}_{s}\left(k,x\right){I}_{r}\left(k\right)}\exp \left[{kL}+k\delta L\bullet x\right]$$

The fundamental contrast for full-range imaging is the phase shift introduced by the reference pathlength modulation. A-line oversampling by a factor of at least two is required to ensure a quasi-static interferogram for effective phase modulation. We used a 1.6 μm step size between A-lines, or equivalently $\delta L$ = 0.013 (*i.e*. 13 μm reference pathlength change per 1 mm fast scanning range). The pathlength modulation is implemented by feeding the HTGM the same ramping voltage waveform as the fast scanning, with an amplitude scaling.

The slow scanning modulation is used to compensate for the large curvature of the retina. We used the empirical non-linear function below to model the posterior curvature along the slow scanning direction and assumed the fovea was at the middle B-scan frame. The voltage was sent to the HTGM to maintain the image placement around the zero delay line,

9 $$L\left(i\right)=\frac{A}{1.5}\log \left(\frac{{10}^{1.5}-1}{\frac{N}{2}-1}\times \left|i-\frac{N}{2}\right|+1\right),i=1,\ldots N$$

where $L\left(i\right)$ was the compensation pathlength at the *i* ^*th*^ frame. *A* was the coefficient for the compensation, equaling to −1.1, and *N* was the total number of B-scan frames.

### Imaging protocols

We prepared multiple scanning patterns and summarized them in Table 1, including the preview and acquisition protocols for Wide Field, Full-range, High Definition, Raster, Circular-Doppler and Angio (in Supplementary Fig. 4) imaging. We set the imaging line rate at 120 kHz (7.5 μs exposure time). The time of all acquisition patterns was 2.18 sec except Angio with 2.56 sec.

**Table 1:** Imaging scan protocols.

	A-scan (n)	B-scan(n)	Scanning Range (mm)	Acquisition Time (s)
Preview (Radial)	512	4	2 × 2	0.02
Wide Field	512	512	13.3 × 13.3	2.18
Full range	8192	32	13.3 × 13.3	2.18
High Definition	2048 × 16	8	6.6	2.18
Raster	512	512	6.6 × 6.6	2.18
Circular-Doppler	8192	32	d:1.65 to d:5	2.18
Angio	360 × 2	384	3.3×3.3	2.56

The preview protocol was a 4-line radial pattern with an angular interval of π/4. Wide field covered an area of 13.3 mm × 13.3 mm with 512 A-lines × 512 B-scans. Full-range imaging protocol was composed of 32 B-scans with equal spacing covering 13.3 mm. In each B-scan, a total of 8192 A-lines in 13.3 mm were scanned. High Definition protocol was implemented to reduce speckle noise and produce high-quality images^37^. In our work, we included 8-line raster and 8-line radial patterns. 16 modulated A-lines over ~0.2 mm orthogonal to the B-scan direction were averaged. The angular interval of the 8-line radial pattern was π/8. All B-scans in the High-Definition protocol have 2048 points. The Circular-Doppler protocol has 32 equally spaced circular B-scans with diameters expanding from 1.65 mm to 5 mm. 8192 A-lines were included in each circular B-scan. For Angio protocol, 360 A-lines × 384 B-scans were acquired twice over 3.3 mm × 3.3 mm.

### OCT and OCTA image processing

The raw data from both channels were processed with general procedures including DC removal, wavelength to wavenumber interpolation (only NIR), and dispersion compensation. We carried out the fast Fourier transform (FFT) to create B-scan images from the absolute values of complex results. For per-A-line noise cancellation, the DC removal used the reference spectrum acquired by the second calibrated/scaled spectrometer. For full-range VIS-OCT imaging, a Hilbert transform was performed along the lateral direction before FFT. For Doppler OCT, the angular value after FFT was extracted, and the phase difference between adjacent A-lines was calculated by subtraction. Then, a 3 × 3 median filter was used to remove the random noise for display.

For OCTA images, we performed the split spectrum method in NIR-OCT using 20 windows with an interval of 7.3 nm to sweep the raw spectra, then processed each swept spectra with the general procedures mentioned above and obtained the complex OCT signals. Between two adjacent complex B-scans, the axial phase group difference was corrected^55^. Then, the differences between consecutive complex B-scans at same locations created one OCTA frame,

10 $${I}_{{OCTA}}\left(x,z\right)=\frac{1}{N-1}{\sum }_{n=1}^{N}\left|{C}_{n+1}^{{NIR}-{OCT}}\left(x,z\right)-{C}_{n}^{{NIR}-{OCT}}\left(x,z\right)\right|$$

in which C(x,z) is the complex B-scan, and N = 2. In each OCTA frame, we applied the maximum intensity projection from the ILM to the ONL to generate the *en face* vasculature images. Finally, all 20 of these *en face* images were averaged to produce the final angiography.

### Human imaging

All the imaging protocols have been approved by the Institutional Review Board (IRB) of Johns Hopkins University School of Medicine. The consent of volunteers was obtained before imaging. The eyes of all subjects were normal but with a low myopia ranging from −1.00 D to −4.00 D.

We first moved the dual-channel light beam onto the iris of subject, then shut off the VIS-channel and used the NIR for the alignment. We asked the subjects to track the illuminated LED dot using the fellow eye for fixation. At the same time, the four frames in the preview mode were continuously refreshing. After the desired locations of the retina were confirmed, we turned on the VIS channel, optimized the focus of the tunable lens, and started the acquisition.

### Reporting summary

Further information on research design is available in the Nature Portfolio Reporting Summary linked to this article.

### Supplementary information

Supplemental Material

Reporting Summary

## Data Availability

The data that support the findings of this study are available from the corresponding author upon reasonable request.

## Code availability

The code that supports the findings of this study is available from the corresponding author upon reasonable request.
