# Supplementary material for: A dual-channel visible light optical coherence tomography system enables wide-field, full-range, and shot-noise limited human retinal imaging
Source: Commun Eng. 2024 Jan 31;3:21. doi: 10.1038/s44172-024-00167-7 (PMC11031604; doi:10.1038/s44172-024-00167-7)
Supplement: Supplementary file 1 — Supplemental Material [file 44172_2024_167_MOESM1_ESM.pdf]

***Supplemental materials**: Second-generation dual-channel visible light optical coherence tomography enables wide-field, full-range, and shot-noise limited retinal imaging*

Jingyu Wang[1,*], Stephanie Nolen[2,*], Weiye Song[3], Wenjun Shao[1], Wei Yi[3], Amir Kashani[1], Ji Yi[1,2,*,†]

1. Department of Ophthalmology, Johns Hopkins University, Baltimore, MD, USA, 21231,
2. Department of Biomedical Engineering, Johns Hopkins University, Baltimore, MD, USA, 21231
3. Department of Medicine, Boston University School of Medicine, Boston Medical Center, Boston, MA, USA ,02118

*These authors contributed equally

†Correspondence: jiyi@jhu.edu

1. Wide field retinal VIS-OCT imaging

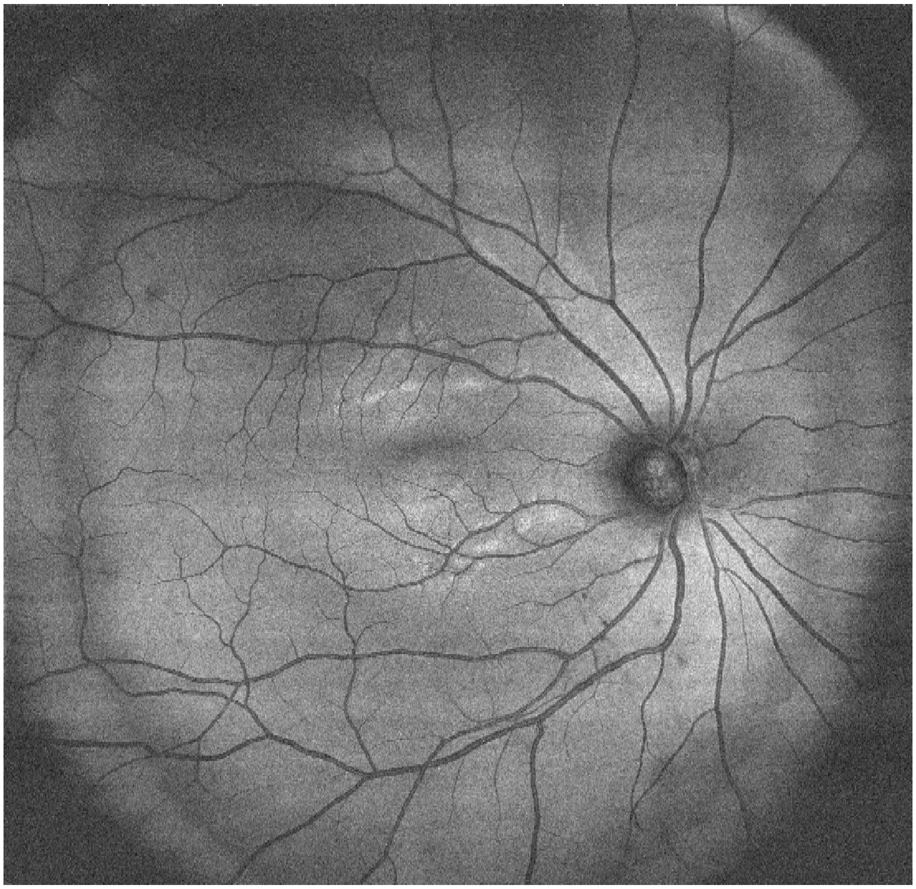

Suppl Fig. 1. *En face* projection of wide-field VIS-OCT imaging >60° viewing angle. The scanning density is 1024x512 acquired at 100kHz Aline rate.

2. Spectrum from visible and near infrared channels.

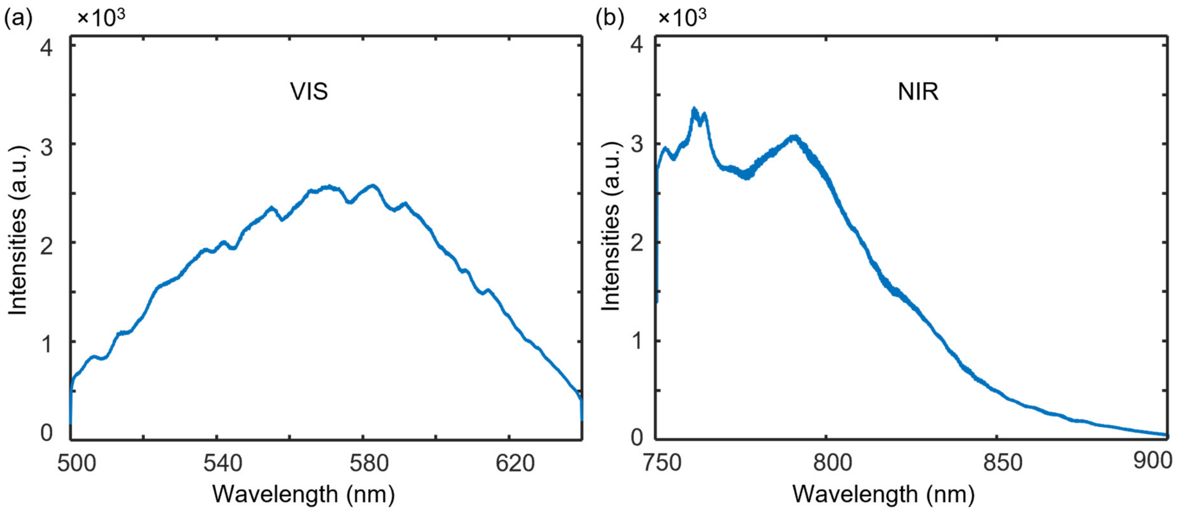

Suppl Fig 2. The raw spectra in VIS-OCT (a) and NIR-OCT (b) channels.

3. Device photograph

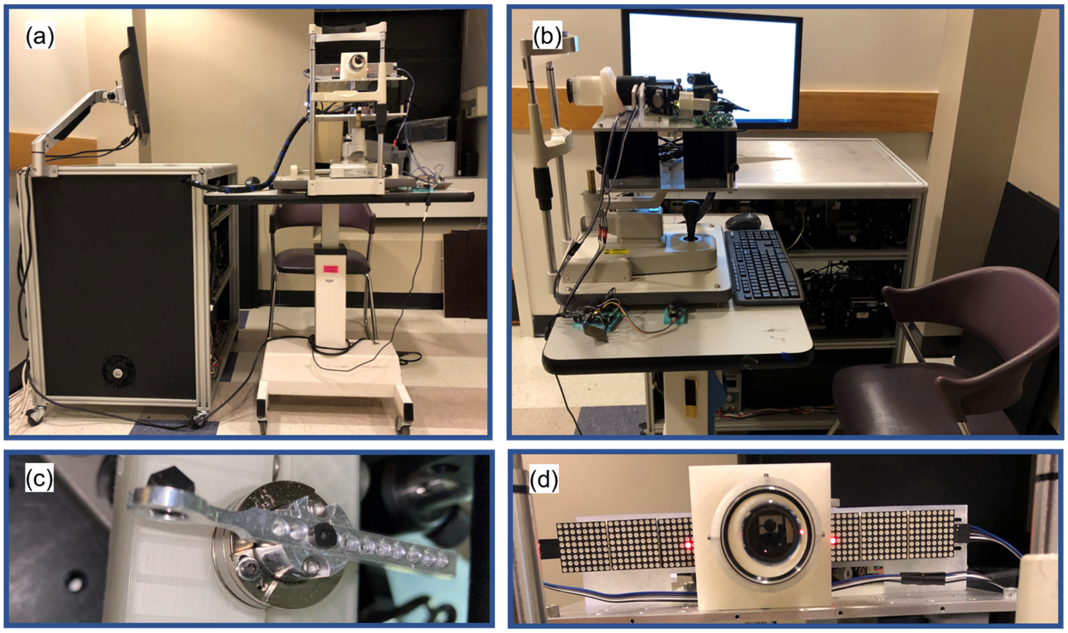

Suppl Fig. 3. Photograph of the device from front and side views(a-b). Photograph of custom-made mounting for the retroreflector on a high-torque galvanometer (HTGM) and the fixation target using LED arrays (c-d).

4. Scanning protocols

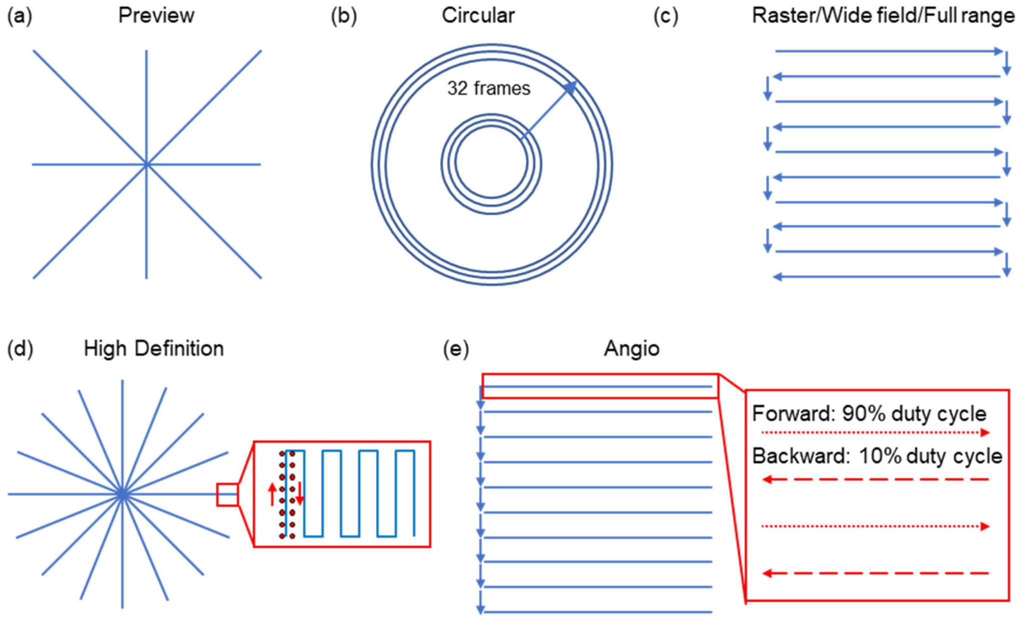

Suppl Fig. 4. Schematic of the scanning path for different protocols encompassed in this paper for the preview (a), circular disc scan (b), raster scan (c), high-definition scan, and (e) angiography scan.

5. Roll-off at different wavelengths.

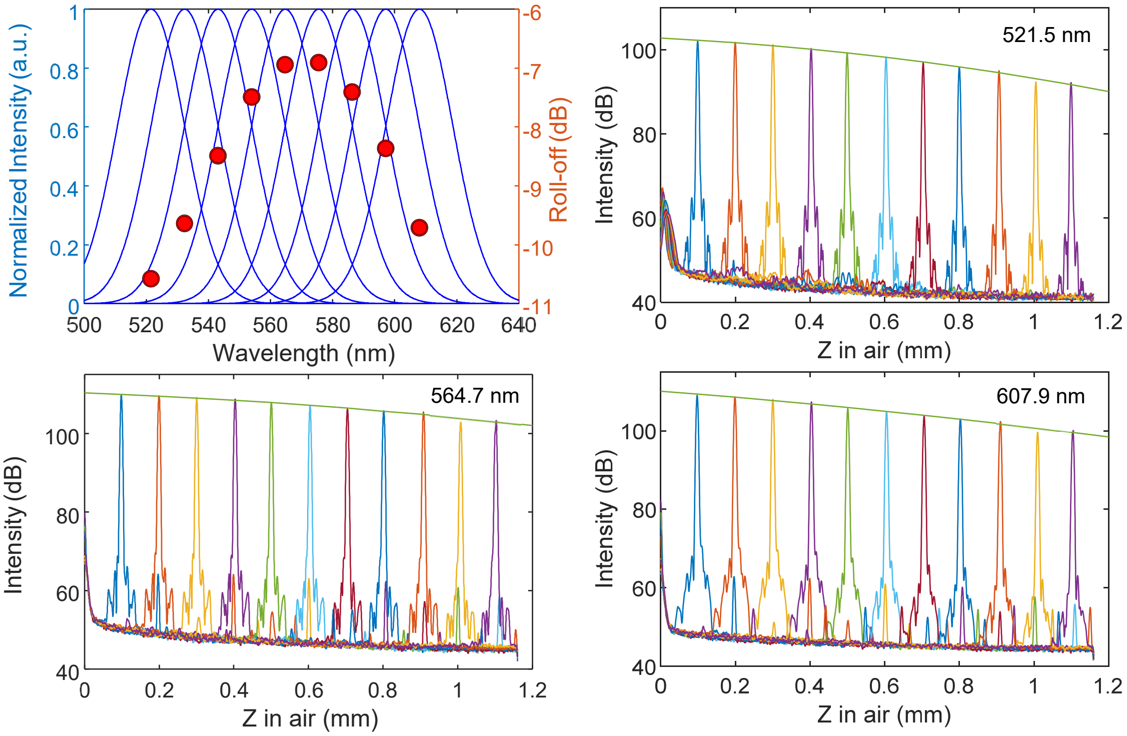

Suppl Fig. 5. Sweeping Gaussian windows and roll-off at different centered wavelengths. (a) Swept wavelength window and roll-off at each wavelength window, (b-d) roll-off of different wavelengths centered at 521.5 nm, 567.7 and 607.9 nm.

6. Roll-off characterization

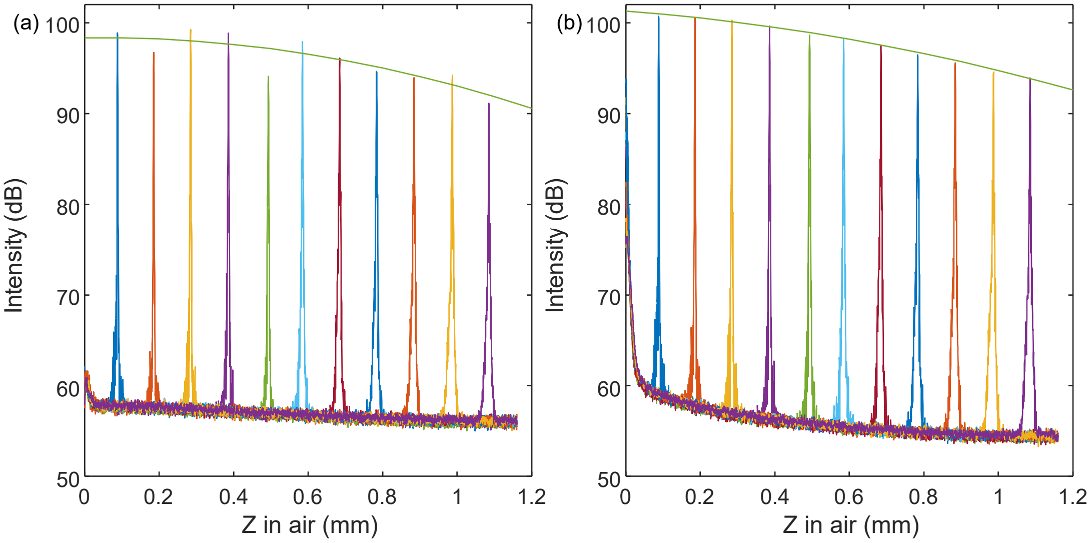

Suppl Fig. 6. Roll-off characterization. (a) With noise cancellation, (b) without noise cancellation.

7. Comparison of correlation matrixes

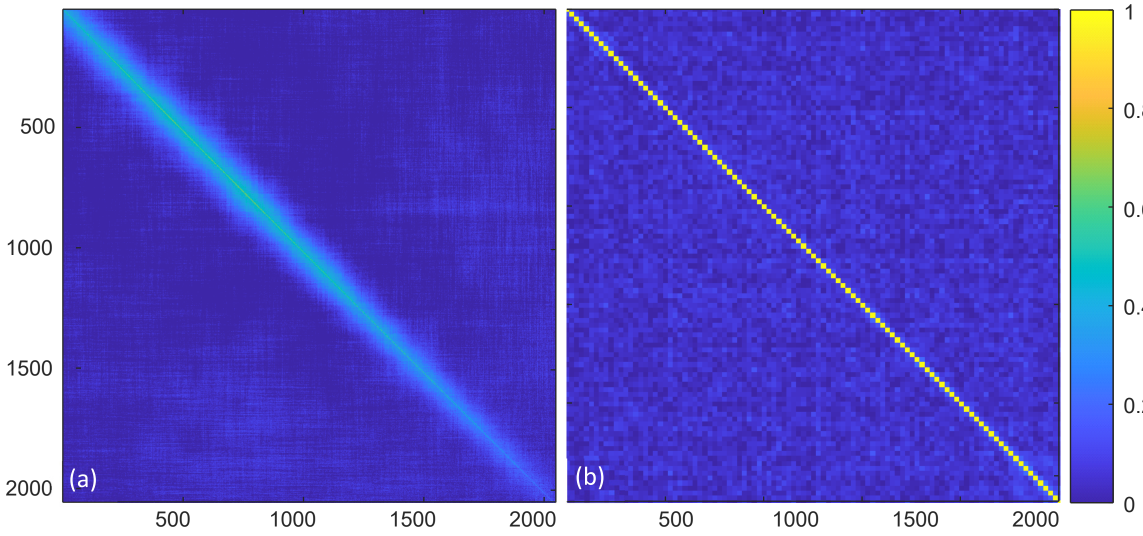

Suppl Fig. 7. Comparison of correlation matrixes: (a) cross correlation matrix between two spectrometers, (b) autocorrelation after noise cancellation.

8. System performance comparison

Supplemental Table 1 Comparison of system performance by different research groups.

Authors	Axial resolution in air (μm)	Roll-off at 1mm in air	Roll-off at entire depth in air(dB)	Full image depth in air(mm)	Line rate (kHz)
Zhang et al[1,2]	1.7	3~4	~14.4*	~2.1	70
Rubinoff et al[3]	1.7	4	~13*	1.5	125
Pi et al[4]	1.7	6	~25*	1.8	50
Ours	1.7	6.2	7.2	1.16	120
*, this value was estimated from original figure.					

Supplementary References

1. Zhang, Tingwei, Aaron M. Kho, and Vivek J. Srinivasan. "Water wavenumber calibration for visible light optical coherence tomography." Journal of biomedical optics 25, no. 9 (2020): 090501-090501.
2. Zhang, Tingwei, Aaron M. Kho, and Vivek J. Srinivasan. "Improving visible light OCT of the human retina with rapid spectral shaping and axial tracking." Biomedical optics express 10, no. 6 (2019): 2918-2931.
3. Rubinoff, Ian, David A. Miller, Roman Kuranov, Yuanbo Wang, Raymond Fang, Nicholas J. Volpe, and Hao F. Zhang. "High-speed balanced-detection visible-light optical coherence tomography in the human retina using subpixel spectrometer calibration." IEEE Transactions on Medical Imaging 41, no. 7 (2022): 1724-1734.
4. Pi, Shaohua, Acner Camino, Miao Zhang, William Cepurna, Gangjun Liu, David Huang, John Morrison, and Yali Jia. "Angiographic and structural imaging using high axial resolution fiber-based visible-light OCT." *Biomedical optics express* 8, no. 10 (2017): 4595-4608.
